# Supplementary figures and images for: The kinesin KIF4 mediates HBV/HDV entry through the regulation of surface NTCP localization and can be targeted by RXR agonists in vitro
Source: PLoS Pathog. 2022 Mar 21;18(3):e1009983. doi: 10.1371/journal.ppat.1009983 (PMC8970526; doi:10.1371/journal.ppat.1009983)

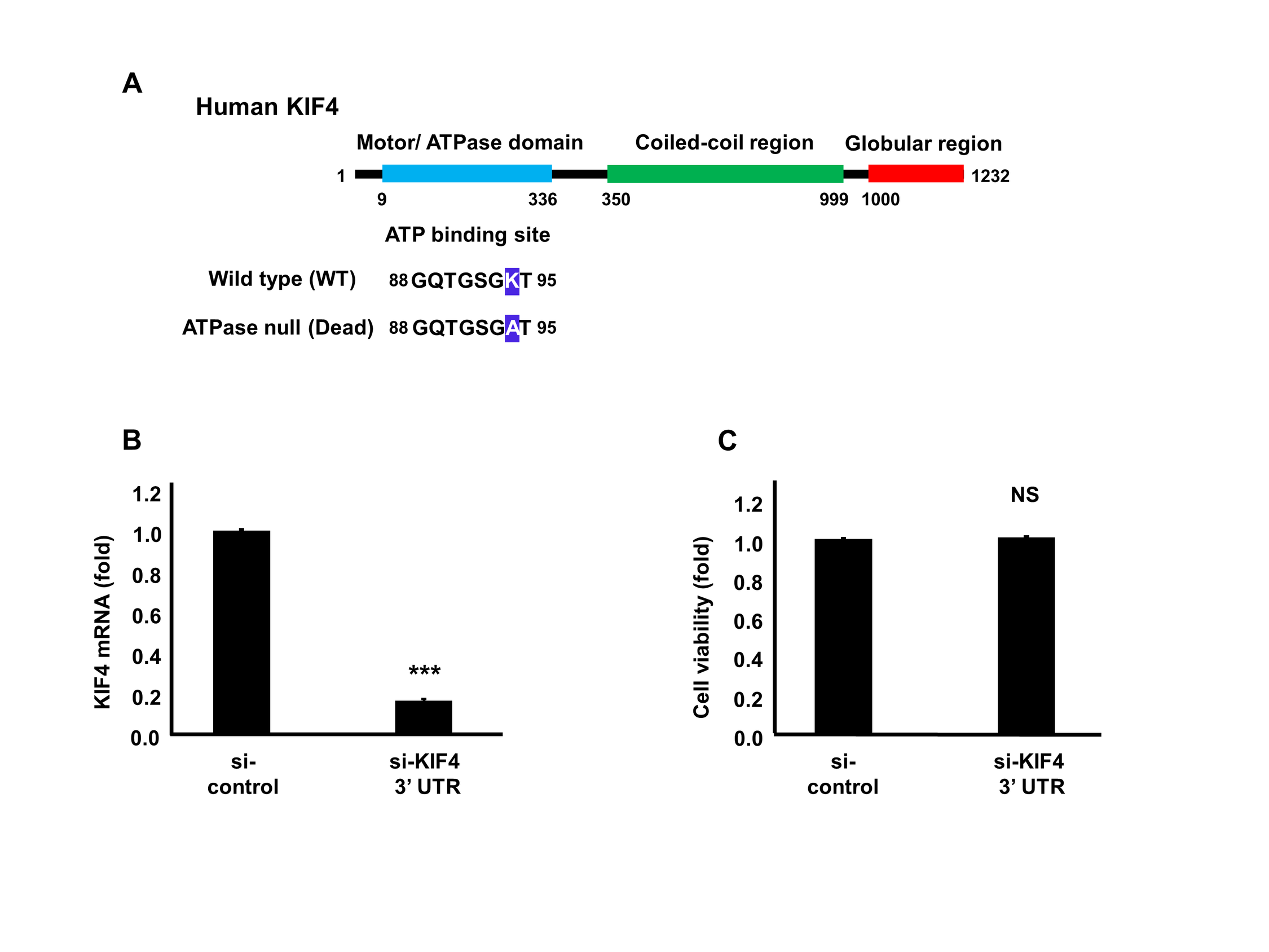

Supplement: S1 Fig — Two sequence alignments show ATP-binding Walker A consensus site in the KIF4 motor domain with lysine 94 (wild-type, upper sequence) was mutated to alanine (ATPase-null motor dead mutant, lower sequence). (B) HepG2-hNTCP were transfected with si-control or si-KIF4 3′ UTR for 48 h; the cells were then lysed and the total RNA content was extracted and the KIF4 expression levels were quantified by RT-qPCR and normalized to the expression of ACTB; or (C) the cell viability was examined using XTT assay. Data are presented as fold changes relative to those of the control siRNA-transfected cells. All assays were performed in triplicate, and data from three independent experiments were included. The data were pooled to assess the statistical significance. Data are presented as mean ± SD. ***, P < 0.001; NS, not significant. (TIF) [file ppat.1009983.s001.tif]

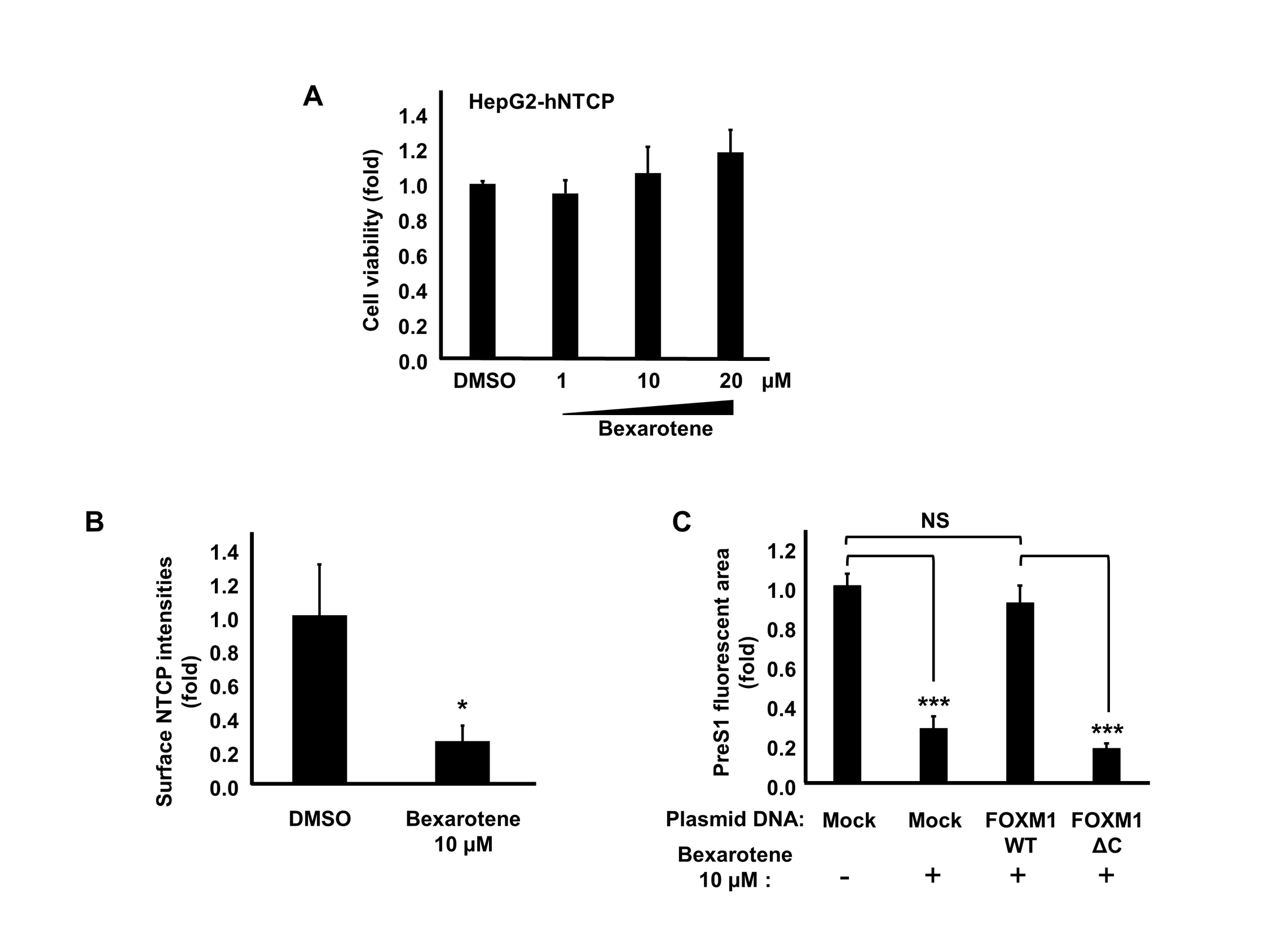

Supplement: S2 Fig — (B) The intensities of surface NTCP bands (normalized to CDH-1) shown in Fig 7B were quantified by ImageJ software and presented as fold changes relative to the control DMSO-treated cells. (C) The fluorescent intensities of preS1 signal shown in Fig 7D were measured and presented as fold changes relative to mock-transfected DMSO-treated cells. All assays were performed in triplicate and data from three independent experiments were included. The data were pooled to assess the statistical significance. For panel (C), the assay was performed in triplicate, and data from two independent experiments were pooled. Data are presented as mean ± SD. *, P < 0.05; ***, P < 0.001; NS, not significant. (TIF) [file ppat.1009983.s002.tif]
