# Supplementary material for: The kinesin KIF4 mediates HBV/HDV entry through the regulation of surface NTCP localization and can be targeted by RXR agonists in vitro
Source: PLoS Pathog. 2022 Mar 21;18(3):e1009983. doi: 10.1371/journal.ppat.1009983 (PMC8970526; doi:10.1371/journal.ppat.1009983)
Supplement: Attachment — Submitted filename: Response to reviewers Final.docx [file ppat.1009983.s003.docx]

**Re: PPATHOGENS-D-21-01953**

**Title: “The kinesin KIF4 mediates HBV/HDV entry through regulation of surface NTCP localization and can be targeted by RXR agonists in vitro.”**

Dear Dr. Jianming Hu:

Associate Editor (Plos Pathogens)

Thank you very much for your e-mail on November the 1^st^. Enclosed we are sending our revised manuscript. In accordance with the reviewers’ suggestions, we have modified our paper and replied to the reviewers’ comments as shown below.

We believe that we have sufficiently revised our paper in accordance with the suggestions of the reviewers and that our revised manuscript is now acceptable for publication.

With our best regards,

Hussein H. Aly, and Takaji Wakita

National Institute of infectious diseases

1-23-1 Toyama, Shinjuku, Tokyo 162-8640, Japan.

Phone +81-3-5285-1111
FAX +81-3-5285-1150

e-mails: [ahussein@niid.go.jp](mailto:ahussein@niid.go.jp), and [wakita@niid.go.jp](mailto:wakita@niid.go.jp)

**Response to Editor Comments**

**Editor’s comments**

Thank you very much for submitting your manuscript "The kinesin KIF4 mediates HBV/HDV entry through regulation of surface NTCP localization and can be targeted by RXR agonists in vitro." for consideration at PLOS Pathogens. As with all papers reviewed by the journal, your manuscript was reviewed by members of the editorial board and by several independent reviewers. The reviewers appreciated the novelty and significance of the work but also raised a number of important issues that should be addressed. In particular, 1- they requested to restore KIF function to KIF KD cells in the context of viral infection, 2- to clarify the mechanisms of bexarotene in relation to what has been reported in the literature vs. in your study, 3- to improve the quality of some of the data (e.g., some western blots), 4- and to use more reliable readout of HBV infection such as HBeAg secretion. Also, as the manipulations used in the study may have pleiotropic effects on viral gene expression and/or antigen secretion, 5- direct measurement of HBV cccDNA as a readout of productive entry (infection) would be helpful in addition to indirect readouts like HBeAg secretion. In light of the reviews (below this email), we would like to invite the resubmission of a significantly-revised version that takes into account the reviewers' comments.

**Response to Editor**

Dear Dr. Jianming Hu,

As for the reviewer’s request (Editor comments 1 to 4)

We addressed these comments in our answer to the reviewers.

As for the Editor’s comment,

5- direct measurement of HBV cccDNA as a readout of productive entry (infection) would be helpful in addition to indirect readouts like HBeAg secretion.

We answered this comment in our reply to reviewer 1, pages 3 to 6 **(in this letter)**, lines 61 to 170.

**Reviewer 1**

**Part I - Summary** 
**Reviewer 1/Q1**

Certainly, the current findings were all based on knock-down rather than knock-out of KIF4. Hence some of the data are not so impressive (such as HBsAg in Fig. 2B; the high titer in both si-NTCP and si-KIF4 cells could be attributed to residual inoculum when each cell was inoculated with 6000 HBV genomes. HBeAg would be a more reliable infectivity marker in that case). That leaves open the question of whether KIF4 is essential or there are redundant pathways for NTCP cell surface localization.

**Reviewer 1/A1**

We wish to thank the reviewer for this comment that help strengthen our results. I agree that we implemented different techniques (ELISA, IF, real-time PCR, Southern blot, and Luciferase) to evaluate the effect of KIF4 on HBV infection. The variable sensitivities of the different methods used is expected to be the reason beyond the fluctuation of the suppressive effect of silencing KIF4 expression on HBV infection. However, in all these experiments we employed the silencing of NTCP expression (HBV-entry receptor) as a positive control; interesting enough silencing of KIF4 expression showed a similar effect on HBV infection to that observed when NTCP silencing was performed.

Following the reviewer and editor comments, we analyzed the effect of siRNA-silencing of KIF4 expression on HBV infection in HepG2-NTCP and Primary Hepatocytes (PXB) by measuring HBV **cccDNA** levels (Fig. 2B and 2H) by real-time PCR, as predicted silencing of KIF4 expression significantly suppressed HBV cccDNA levels in HepG2-NTCP (*P* < 0.01), and primary human hepatocytes (*P* < 0.01). The suppressive effect of silencing KIF4 expression on HBV infection in primary hepatocytes was also confirmed by measuring **HBeAg** levels (*P* < 0.001) (Fig. 2G).

To add these changes to the paper we added

**Results (Lines 139 to 156)**

We added the underlined text below:

**(KIF4 is required for cell culture-derived HBV (HBVcc) infection)**

We used authentic HBV particles generated from grown HepAD38.7-Tet cells to validate the relevance of KIF4 in the HBV life cycle (HBVcc). We reduced KIF4 expression in HepG2-hNTCP cells by transfecting particular siRNA 72 hours before HBVcc infection (Fig. 2A); after 10 to 13 days post infection (pi), we examined its influence on hepatitis B surface antigen (HBsAg), cccDNA, DNA, and HBc levels. As a positive control, si-NTCP was utilized. Silencing KIF4 expression resulted in a substantial decrease of HBsAg in the culture supernatant (*P* < 0.001) and HBV cccDNA (*P* < 0.01) to levels equivalent to silencing NTCP expression (Fig. 2B). siKIF4 also decreased total HBV DNA levels as measured by southern blot (Fig. 2C) and inhibited HBc expression as measured by immunofluorescence (IF) (Fig. 2D) to levels equivalent to si-NTCP without compromising cellular viability (Fig. 2E). We employed primary human hepatocytes (PXB) cells to examine the effect of suppressing KIF4 expression on HBV infection in a more physiologically relevant paradigm (Fig. 2F). As predicted, inhibiting KIF4 or NTCP expression dramatically reduced HBsAg (*P* < 0.01) and HBeAg (*P* < 0.001) levels. (Fig. 2G) and decreased extracellular HBV DNA (*P* < 0.001) and HBV cccDNA (*P* < 0.01) levels as detected by real-time PCR. (Fig. 2H). Figure 2I depicts the silencing efficiency of KIF4 siRNA in PXB cells.

**To the figures**

We added the quantification of **HBV cccDNA** levels to Fig .2B; and Fig. 2H.

We also added the quantification of **HBeAg** levels to Fig. 2G.

**To the figure legends**

We added the underlined text below to Figure Legend 2 (**lines 662 to 694**)

**Decreased KIF4 expression suppressed HBV infection in HepG2-hNTCP and primary human hepatocytes (PHH).**

**(A)** Schematic diagram depicting the scheme for siRNA transfection and subsequent HBV infection in HepG2-hNTCP; HepG2-hNTCP cells were transfected with si-control, si-NTCP, or si-KIF4 (si-1) for 72 h and then inoculated with HBV at 6,000 GEq/cell in the presence of 4% PEG8000 for 16 h. After free HBV were washed out, the cells were cultured for an additional 12 days, followed by the detection of different HBV markers. Black and dashed boxes indicate the interval for treatment and without treatment, respectively. **(B)** HBsAg secreted into the culture supernatant was collected at 10 dpi, quantified by ELISA, and presented as fold changes, relative to the values of control siRNA transfected cells. **(B)** HBV cccDNA, **(C)** Intracellular HBV DNA, and **(D)** HBc protein in the cells were detected at 13 dpi by real-time PCR, Southern blot analysis, and immunofluorescence, respectively. Red and blue signals in panel **(D)** depict the staining of HBc protein and nucleus, respectively. **(E)** Cell viability was also examined by the XTT assay. **(F)** Schematic diagram showing the scheme for siRNA transfection and the subsequent HBV infection in primary human hepatocytes (PXB); primary human hepatocytes were twice transfected with si-control, si-NTCP, or si-KIF4 (si-1) for consecutive 72 h and 48 h, followed by HBV inoculation at 1,000 GEq/cell in the presence of 4% PEG8000 for 16 h. After being washed, the cells were cultured for an additional 15 days. **(G)** HBsAg and HBeAg secreted into the culture supernatant were quantified by ELISA and Chemiluminescent Immuno‐Assay, respectively, and the data were presented as fold changes, relative to the values of control siRNA-transfected cells. (**H**) Extracellular HBV DNA in the culture supernatant and HBV cccDNA (extracted at 8 dpi) were quantified by real-time PCR. In all infection assays including siRNA transfection, control siRNA and NTCP-targeting siRNA were used as negative and positive controls, respectively. (**I**) Primary human hepatocytes were twice transfected with siRNAs (as shown in Fig. 2 F); the total protein was extracted and KIF4 (*upper panel*) and β-actin (loading control) (*lower panel*) expression were analyzed by immunoblotting with the respective antibodies. All assays were performed in triplicate and included 3 independent experiments. Standard deviations are also shown as error bars*.* For HBV cccDNA in panel **(H)**, the assay was performed in triplicate, and data from 2 independent experiments were pooled (n=6). Statistical significance was determined using Student’s *t-*test (**, *P* < 0.01; ***, *P* < 0.001; NS, not significant).

**To Material and Methods, we added the underlined text below.**

**lines 576 to 583**

**DNA and RNA extraction**

Intracellular HBV DNA and HBV cccDNA were extracted from the cells using the QIAamp Mini Kit (QIAGEN). For HBV cccDNA, DNA extraction was performed without proteinase K treatment as previously described (1). Extracellular HBV DNA was recovered from the supernatant using the SideStep Lysis and Stabilization Buffer (Agilent Technologies, 400900), while RNA extraction was performed using the NucleoSpin® RNA XS Kit (MACHEREY-NAGEL) according to the manufacturer’s protocols.

**Lines 587 to 609**

**qPCR and RT-qPCR**

Real-time PCR (for detection of total HBV DNA and HBV cccDNA) and reverse transcription real-time PCR (for the measurement of HDV RNA) were essentially performed as previously described (1-3) using the primer-probe sets; 5′-AAGGTAGGAGCTGGAGCATTCG-3′, 5′-AGGCGGATTTGCTGGCAAAG-3′, 5′-FAM-AGCCCTCAGGCTCAGGGCATAC-TAMRA-3′ for HBV DNA, 5′-GTGGTTATCCTGCGTTGAT-3′, 5′-GAGCTGAGGCGGTATCT-3′, 5′-FAM-AGTTGGCGAGAAAGTGAAAGCCTGC-TAMRA-3′ for HBV cccDNA and 5′-GGACCCCTTCAGCGAACA-3′, 5′-CCTAGCATCTCCTCCTATCGCTAT-3′, 5′-FAM-AGGCGCTTCGAGCGGTAGGAGTAAGA-TAMRA-3′ for HDV RNA. qPCR for Intracellular HBV DNA and HBV cccDNA were performed by the 2^(−ΔΔCT)^ method using chromosomal GAPDH DNA sequence (via primer-probe set Hs04420697_g1; Applied Biosystems) as an internal normalization control. Isolated RNA was reverse-transcribed using the High-Capacity cDNA Reverse Transcription Kit (Thermo Fisher Scientific), and the relative levels of the KIF4 mRNA were determined using the TaqMan Gene Expression Assay with the primer-probe set Hs00602211_g1 (Applied Biosystems), while the ACTB expression (primer-probe set 748 Hs99999903_m1) was included as an internal control for normalization (1).

**HBsAg and HBeAg quantification:**

Cell supernatants were harvested and ELISA quantification of the secreted HBs was performed as described previously (2). The half-maximal inhibitory concentration (IC_50_) value for Bexarotene was calculated as previously reported (4). HBe antigen was quantified by a Chemiluminescent Immuno‐Assay as described previously (5).

**Reviewer 1/ Q2**

Similarly, since the authors demonstrated that silencing endogenous KIF4 coupled with transfection with wild-type but not K94A mutant could restore NTCP cell surface localization, it would be extremely helpful to perform infection experiments on top of that, thus further proving a role of KIF4 in sustaining HBV infectivity through its motor activity.

**Reviewer 1/ A2**

This is an important comment which was shared also by **Reviewer 3**, which will show the functional significance of KIF4 Kinesin motor function on HBV infection.

We followed the reviewer’s comment and performed HBV infection experiment in HepG2-hNTCP cells in which the expression of endogenous KIF4 was silenced by specific siRNA and compensated by exogenously transfecting plasmids expressing si-RNA resistant WT-KIF4 or K94A ATPase-dead mutant. PreS1 binding and HBV infection was evaluated by IF detection of PreS1-Tamra and HBc respectively. As expected, WT but not ATPase-dead KIF4 increased the susceptibility to PreS1 binding and HBV infection in endogenous KIF4-silenced cells.

**Result section, lines 193 to 215**

We added the highlighted text below.

**(KIF4 motor activity is required for surface NTCP expression**

Kinesins, such as KIF4, are motor proteins that hydrolyze ATP to transport different molecules along microtubules (6). Because KIF4 is necessary for surface NTCP expression, we hypothesized that it may function as a transporter that delivers NTCP to the cell surface. As a result, we investigated the function of KIF4 ATPase activity in NTCP surface expression. The sequence of an ATPase-null KIF4 was previously described (7) (Fig. S1A). To mute endogenous KIF4 expression, we utilized a tailored siRNA sequence (si-KIF4 3′ UTR) that targeted the 3′ UTR region of the KIF4 transcript (8) and compensated for this suppression by transfecting plasmids expressing the Myc-tagged WT or ATPase-null KIF4 sequences. Because they lack the 3′ UTR of endogenous KIF4 transcripts, the mRNAs of these constructs are resistant to si-KIF4 3′ UTR. Cellular fractionation revealed that the expression of WT or ATPase-null mutants did not affect surface cadherin (CHD-1) expression, as predicted. Surface NTCP levels are considerably enhanced when KIF4 depletion is compensated with WT, but not ATPase-null KIF4 protein (Fig. 5A). These findings were supported by IF analysis, which revealed significantly greater levels of surface NTCP (Fig. 5B), higher binding of HBV preS1 to HepG2-NTCP cells (Fig. 5C), and higher HBV infectivity as detected by HBc levels (Fig. 5D) when endogenous KIF4 silencing was compensated for by WT-KIF4, but not by ATPase-null KIF4. The si-KIF4 3′ UTR exhibited a substantial reduction of KIF4 by real-time RT-PCR and no cellular cytotoxicity as determined by the XTT assay (Fig. S1B and S1C). Overall, our findings indicated that KIF4 ATPase (motor) activity is necessary for NTCP surface expression (transport), and in turn NTCP-mediated HBV binding and infection.)

**Figures, we changed Figure 5**

Previous Figure 5A, D and E were moved to the supplementary figure 1A, B and C respectively.

Previous Figure 5B became 5A

Previous Figure 5C became 5B.

A new panels 5C and 5D were added to the paper showing the effect of motor active, but not ATPase-dead KIF4 on the permissiveness to HBV infection in cells in which endogenous KIF4 was suppressed by specific siRNA.

Supplementary Figure 1 was added

**Figure Legends,**

**Changes to figure legend 5 (lines 749 to 783) were underlined below);**

**(KIF4 motor activity is required for the surface NTCP expression.**

**(A)** HepG2 cells were transfected with si-KIF4 3′ UTR (targeting endogenous KIF4 mRNA 3` UTR region) along with HA-NTCP, and Myc-KIF4 (WT or ATPase-null) plasmid vectors for 72 h; the cells were then surface biotinylated or PBS treated at 4°C for 30 min before cell lysis. After centrifugation and the removal of cell debris, cell lysates were collected and an aliquot (1/10 volume) was used for the detection of HA-NTCP (*Input;* *upper panel*), Myc-KIF4 (*Input; middle panel*), and β-actin (loading control) (*Input; lower panel*) by immunoblotting. The remaining cell lysates (9/10 of the original volume) were subjected to pull-down via incubation with pre-washed SA beads for 2 h at 4°C; after being washed, the biotinylated surface proteins were eluted and subjected to western blotting in order to detect surface HA-NTCP (*Surface; upper panel*) and CDH-1 (loading control for surface fraction) (*Surface; lower panel*) with the respective antibodies. **(B)** HepG2-hNTCP were transfected with si-KIF4 3′ UTR and Myc-KIF4 WT (*upper panel*) or motor dead mutant (*lower panel*) plasmid vectors; at 3 days post-transfection, the cells were fixed, permeabilized, stained with the indicated antibodies, and visualized by confocal microscopy. Green, red, and blue signals represent the staining of NTCP, Myc-KIF4, and nuclei, respectively. The arrow heads show NTCP localization in the WT or ATPase-null KIF4 transfected cells. **(C)** HepG2-hNTCP were co-transfected with si-KIF4 3′ UTR and Myc-KIF4 WT (*upper panel*) or motor dead mutant (*lower panel*) plasmid vectors; at 3 days post-transfection, the cells were incubated with TAMRA-labeled preS1 peptide (preS1 probe) for 30 min at 37°C; the cells were then washed of free preS1 probe, fixed, permeabilized, stained with the indicated antibodies, and visualized by confocal microscopy. Red, Green, and blue signals represent preS1 probe, Myc-KIF4, and nuclei, respectively. The arrow heads show preS1 surface localization. **(D)** HepG2-hNTCP were co-transfected with either si-control or si-KIF4 3′ UTR, and Myc-KIF4 (WT or ATPase-null) expression vectors or empty vector for 72 h; the cells were then inoculated with HBV at 6,000 GEq/cell in the presence of 4% PEG8000 for 16 h. After washing free HBV particles, the cells were cultured for an additional 12 days, followed by the examination of HBc protein (*upper panel*) in the cells by IF. Red and blue signals show the staining of HBc protein and nucleus, respectively and HBc fluorescence intensities are shown in the graph (*lower panel*). All assays were performed in triplicate and data from 3 independent experiments were included. For panel **(D)**, the assay was performed in triplicate and, data from two independent experiments were pooled (n=6). The data were pooled to assess the statistical significance. Data are presented as mean ± SD. ***, *P* < 0.001; NS, not significant.)

**Supplementary Figure 1 was added (lines 907 to 919)**

**(Fig S1**

**(A)** A schematic diagram illustrating human KIF4 domains and the key regions are presented at the top of the figure. Two sequence alignments show ATP-binding Walker A consensus site in the KIF4 motor domain with lysine 94 (Wild type, *upper sequence*) was mutated to alanine (ATPase-null motor dead mutant, *lower sequence*). **(B)** HepG2-hNTCP were transfected with si-control or si-KIF4 3′ UTR for 48 h; the cells were then lysed and the total RNA content was extracted and the KIF4 expression levels were quantified by RT-qPCR and normalized to the expression of ACTB; or **(C)** the cell viability was examined using XTT assay. Data are presented as fold changes, relative to those of the control siRNA-transfected cells. All assays were performed in triplicate and data from 3 independent experiments were included. The data were pooled to assess the statistical significance. Data are presented as mean ± SD. ***, *P* < 0.001; NS, not significant. )

**Discussion, lines 320 to 322** the highlighted text below was added

(a phenomenon that was accompanied by a significant suppression of HBV infectivity (Fig. 5).)

**Part II – Major Issues: Key Experiments Required for Acceptance**

**Reviewer #1 Q3:**

**Some figures of Western blot are of poor quality, such as Fig. 1E, top panel, Fig. 5B, top panel, and Fig. 7B, top panel.**

**Reviewer #1 A3:**

This is an essential comment to improve the quality of our work. We followed the reviewer comment and new western blot analysis using lysates from the same experiments that were stocked at -80 C were used at Fig. 1E, 5B and 7B.

**Please see Figures 1E, 5A (instead of 5B), and 7B.**

**Part III – Minor Issues: Editorial and Data Presentation Modifications**
Please use this section for editorial suggestions as well as relatively minor modifications of existing data that would enhance clarity.

**Reviewer #1 Q4:**

1. The authors cited and interpreted data from Dr. Wenhui Li’s group (ref. 28). According to that paper, in which Bexarotene was added during but not prior to HBV infection, it did not alter NTCP cell surface localization. What's the explanation for the discordance?

**Reviewer #1 A4:**

We thank the reviewer for this nice comment. In the paper described by Dr. Winhui Li’s group, in supplementary figure 2, The effect of Bexarotene treatment on NTCP expression was only evaluated at the mRNA level, these data are actually in accordance with our conclusion that Bexarotene did not affect total NTCP expression but affected the transport of NTCP to cell surface (surface NTCP protein level). Furthermore, Dr. Li’s group showed that silencing of RXRα did not affect surface NTCP proteins levels. There are three known subtypes of the RXR family in humans: RXRα, RXRβ, and RXRγ. Bexarotene is a pan-RXR agonist (9), the paper of Dr. Li’s group focused on RXRα based on its abundancy in hepatocytes, but this does not exclude the possibility that Bexarotene suppresses NTCP expression through its effect on other RXR subtypes.

To reflect these comments in the manuscript, we added to **Discussion** (**lines 347 to 372)** the underlined text below:

(Bexarotene has previously been shown to inhibit the early stages of HBV infection when co-inoculated with HBV during the first 24 hours by Song *et al.* (10). This impact was influenced in part by RXR-regulated gene expression in arachidonic acid (AA)/eicosanoid biosynthesis pathways, which included the AA synthases phospholipase A2 group IIA (PLA2G2A). The specific step of the HBV life cycle (from attachment to cccDNA formation) impacted by Bexarotene was not defined in that study (10) Furthermore, silencing PLA2G2A expression marginally alleviated Bexarotene’s inhibitory impact on HBV infection(10), indicating the presence of other key Bexarotene-dependent mechanisms that are still inhibiting the early stages of HBV infection. In accordance with our data, the effect of Bexarotene treatment on NTCP expression was also evaluated at the mRNA level in this work and showed no effect on total NTCP expression. Although it appears contradictory to our findings, Song *et al.* also showed that silencing of RXRα did not affect surface NTCP proteins levels. However, there are three known subtypes of the RXR family in humans: RXRα, RXRβ, and RXRγ; Bexarotene is a pan-RXR agonist (10) and that mentioned study focused on RXRα based on its abundancy in hepatocytes, but this does not exclude the possibility that Bexarotene suppresses NTCP expression through its effect on other RXR subtypes . In line with Song *et al.* study (10), we found that co-treatment of Bexarotene with HBV inoculation moderately suppressed HBV infection, however, we also showed that the major suppression of HBV infection was obtained when Bexarotene was administrated pre-infection (Fig. 8E and F) suggesting the presence of other significant mechanism by which Bexarotene exerts its suppressive effect on HBV infection. Furthermore, we found that Bexarotene treatment effectively suppressed surface NTCP levels (Fig. 7B), and NTCP bile salts transporter activity (Fig. 7G). Hence, our data clearly showed that the main mechanism by which Bexarotene suppressed HBV infection is through the downregulation of surface NTCP levels prior to HBV infection (Fig. 9). )

**Reviewer #1 Q5/A5:**

2. Some writing issues.

**Line 53**: please replace “immunofluorescence (IF)” with “IF”

We changed the new Manuscript **page 2, Line 54**, it becomes: IF

**Line 84**: “nucleocapsid infects human hepatocytes” should be changed to “nucleocapsid enters human hepatocytes”.

We changed the new Manuscript **page 4, Lines (83-84),** it becomes: When HBV infects human hepatocytes, its nucleocapsid is carried to the nucleus

**Line 186**: “encouraged its accumulation” is better changed to “promoted its accumulation”.

We changed the new Manuscript **page 10, Line 186**, it becomes: promoted its accumulation

**Reviewer #2:**

**Part I - Summary**

Reviewer #2: In this manuscript, Gad et al. have performed functional siRNA screening using HepG2-hNTCP cell-based infection system, aiming to find host factors that can be therapeutically addressed to control HBV infection. The authors identified KIF4, an ATP-dependent microtubule-based motor protein, as a facilitating host factor for HBV/HDV infection. They provided several lines of evidence showing that KIF4 is a critical regulator for cell surface transport of NTCP. They further demonstrated that small molecules downregulating KIF4 expression, e.g. RXR agonist Bexarotene, can suppress HBV infection and be considered as potential antiviral candidates for HBV/HDV infection.
Overall, this manuscript is well written and the in vitro data illustrating a significant role of KIF4 in transporting NTCP to the cell surface for HBV/HDV infection are convincing. This study has made an important contribution to our understanding about how NTCP is transported to the cell surface, however, whether KIF4-targeted therapy using RXR agonists is a practical approach to inhibit HBV/HDV infection in clinical practice need to be further explored. Below are the concerns and suggestions to be addressed

**Part II – Major Issues: Key Experiments Required for Acceptance**

**Reviewer #2 Q1**
**1. The authors stated that “RXR agonists (Bexarotene, and Alitretinoin) down-regulated KIF4 expression via FOXM1-mediated suppression” in the abstract (page 2, line 56), however, experimental data supporting this statement is quite limited. They only showed that FOXM1 and KIF4 protein levels were both decreased in 10 uM Bexarotene-treated cells (Fig. 7C). To further confirm the role of FOXM1 in RXR agonist-mediated blocking of HBV/HDV entry, more experiments should be performed, e.g. rescue experiments using exogenously expressed FOXM1 or FOXM1 dominant negative mutant.**

**Reviewer #2 A1**

We wish to thank our reviewer for this good comment shared also with **Reviewer 3**. As KIF4 expression was previously reported to be regulated by FOXM1 isoform C in HCC (11), We followed the reviewer’s advice. We added Fig. 7D and 7E. Fig.7D shows that Bexarotene (10 uM) treatment suppressed HBV preS1 binding to surface NTCP and exogenously expressed WT FOXM1 isoform C but not the dominant negative mutant (FOXM1-ΔC) relieved Bexarotene suppressive effect on HBV preS1 binding to surface NTCP. These data are parallel to the suppressive effect of Bexarotene on endogenous KIF4 expression shown in Figure 7E second lane, which was also reversed by the exogenous overexpression of WT FOXM1 but not FOXM1-ΔC (third and fourth lanes). Together with the suppressive effect of Bexarotene on NTCP surface expression (showed in Fig. 7B) and HBV/HDV infection (shown in Fig. 8), the data clarify the role of FOXM1 in mediating Bexarotene suppression of KIF4 expression and the resultant impairment of KIF4-mediated surface transport of NTCP and consequent NTCP-mediated HBV/HDV infection.

To the manuscript we changed the following

**Results Section**

We added the underlined text below **(lines 264 to 269)**.

**(RXR agonists down-regulate KIF4 expression and block HBV entry by FOXM1 -mediated suppression**

Consequently, overexpression of WT FOXM1 but not the dominant negative mutant (FOXM1-ΔC) rescued the preS1 binding to surface NTCP (Fig. 7D) and KIF4 expression levels (Fig. 7E) in cells pretreated with 10 µM Bexarotene. As an expected result to the suppression of preS1/NTCP binding by Bexarotene, we found that the pretreatment of HepG2-NTCP with 10 µM Bexarotene significantly suppressed HBV/NL infection in HepG2-NTCP cells (P < 0.001) (Fig. 7F) without altering cell viability (Fig. S2A).)

**Figures**

We added new panels 7D and 7E.

**Figures legends**

We added in Figure 7, (**lines 836 to 847)**

**((D)** HepG2-hNTCP were transfected with HA-tagged FOXM1 WT, HA-tagged FOXM1ΔC dominant negative mutant vectors, or empty vector. 8 h post-transfection, the cells were treated with DMSO or 10 μM Bexarotene for 72 h and then incubated with TAMRA-labeled preS1 peptide (preS1 probe) for 30 min at 37°C and examined by fluorescence microscopy. Red and blue signals indicate preS1 probe and the nucleus, respectively. **(E)** HepG2-hNTCP were transfected with HA-tagged FOXM1 WT, HA-tagged FOXM1ΔC dominant negative mutant vectors, or empty vector. 8 h post-transfection, the cells were treated with DMSO or 10 μM Bexarotene for 72 h and then lysed and total cell lysates were subjected to immunoblotting to detect the protein expression levels of endogenous KIF4 (*upper panel*), ectopically expressed WT or mutant HA-FOXM1 (*middle panel*), and β-actin (loading control) (*lower panel*) with their corresponding antibodies.)

**Material and Methods.**

**Plasmids and siRNA (lines 464 to 475)**

(N-terminally HA-tagged FOXM1 WT and FOXM1ΔC [lacking C-terminal transactivation domain, encoding amino acids 1–616, and serving as dominant negative mutant (12)] constructs were created by PCR amplification of the sequence encoding FOXM1 using HaloTag FOXM1 isoform C expressing plasmid (pFN21AB6289, Promega) as the template; The resulting amplification products were digested by *Xho*I and *Xba*I and subcloned into *Xho*I/ *Xba*I digested pcDNA3.1 (Invitrogen); the primers used in PCR amplification included forward primer 5′-CGGCCGCTCGAGATGTACCCATACGATGTTCCAGATTACGCTAAAACTAGCCCCCGTCGGCC-3′ for both constructs and reverse primers 5′-GGGCCCTCTAGACTACTGTAGCTCAGGAATAAACTGGG-3′ and 5′-GGGCCCTCTAGACTAGACAGATTTGCTCGGGGTGGAGG-3′ for HA-FOXM1 WT and HA-FOXM1ΔC respectively.)

**Reviewer #2 Q2.**

**Co-i**mmunoprecipitation study cannot definitely prove the physical contact of KIF4 and NTCP (page 12, line 223; page 16, line 309). An in vitro pull down assay using purified recombinant KIF4 and NTCP proteins should be performed to rule out the possibility that these two proteins are co-immunoprecipitated through a third partner present in the lysates of cells co-transfected with KIF4- and NTCP-expressing plasmids.

**Reviewer #2 A2.**

This is one of the most important comments that improved the scientific value of our manuscript significantly. In-fact, the reviewer speculation is correct. We followed the reviewer comments and prepared both recombinant KIF4 and NTCP proteins. After co-incubation of the recombinant proteins together at 4 ^o^C, we could not co-immunoprecipitate NTCP with KIF4 despite the successful pulling down of the recombinant KIF4 protein (Fig. 6C). Based on these data we agree that the interaction between KIF4 and NTCP might be mediated by a third partner. In fact, KIF4 was previously reported to regulate the surface expression of L1 adhesion molecule through the participation in the antiretrograde transport of vesicles containing L1 to the axon shaft of neuronal cells on the microtubules (13).

Based on these findings, we added the following

**Abstract** (line 56)

(KIF4 interacts with NTCP )

**Results**

**Interaction between KIF4 and NTCP**

**lines 230 to 240**

(To further investigate the interaction between these two proteins, we prepared recombinant KIF4 and NTCP proteins, after co-incubation of these proteins together, we performed a co-immunoprecipitation experiment by pulling down the Myc-tagged KIF4. Despite the successful pulling down of KIF4, we could not detect the co-precipitation of the recombinant NTCP protein (Fig. 6C). Overall, our findings suggest that KIF4 binds to NTCP possibly indirectly through a complex formation across the microtubules in the cytoplasm utilizing its ATPase motor domain to transport NTCP to the cell surface. In fact, KIF4 was previously reported to regulate the surface expression of L1 adhesion molecule through the participation in the antiretrograde transport of vesicles containing L1 to the axon shaft of neuronal cells on the microtubules (13).)

**Figures**

We added Fig. 6C.

**Figure Legends**

Figure 6 **(lines 804 to 813)**

((C) Recombinant NTCP and KIF4 proteins were co-incubated in PBS (in rotation) at 4C for 8 hours; an aliquot of the mixture (1/10 volume) was used for the detection of Myc-KIF4 (*Input*; *upper panel*), NTCP (*Input*; *lower panel*), by immunoblotting. The remaining protein mixture (9/10 of the original volume) were subjected to co-IP using either isotype control antibody or anti-Myc IgG to pull down Myc-KIF4. Following IP, each sample was analyzed by immunoblotting for Myc-KIF4 (*IP*; *upper panel*) and NTCP (*IP*; *lower panel*). All assays were performed in triplicate and data from 3 independent experiments were included. For panel **(C)**, the assay was performed in duplicate and data from 3 independent experiments were included.)

**Material and Methods (lines 559 to 575)**

(**Recombinant proteins:**

Myc-tagged KIF4 recombinant protein was translated from pIRESpuro3 expression vector using the TNT Coupled Reticulocyte Lysate Systems (Promega, L4611) following manufacturer’s recommendation; while the recombinant NTCP protein preparation was previously reported (14).)

**Co-Immunoprecipitation (Co-IP) assay**

293FT cells were transfected with HA-tagged NTCP and Myc-tagged KIF4 expression plasmids at a 1:1 ratio for the assessment of the possible physical interaction between NTCP and KIF4. At 72 h after transfection, the cells were lysed and subjected to immunoprecipitation with the mouse monoclonal anti-Myc (Santa-Cruz) antibody or mouse normal IgG as a negative control. Following IP, the samples were subjected to immunoblotting to detect co-immunoprecipitated HA-NTCP and α-tubulin (microtubule marker) in the pull-down fraction. Cell lysis and Co-IP were conducted using the Pierce Co-IP Kit (Thermo Fisher Scientific, 26149) according to the manufacturer’s instructions. Recombinant NTCP and KIF4 proteins were co-incubated in PBS at 4C for 8 hours; Co-IP were conducted using the Pierce Co-IP Kit (Thermo Fisher Scientific, 26149) according to the manufacturer’s instructions.)

**Reviewer #2 Q3.**

Many HBV/HDV entry inhibitors would interfere normal physiological function of NTCP, mainly bile acid transportation. The authors should examine the effect of RXR agonists on bile acid transporter function of NTCP in vitro and in vivo.

**Reviewer #2 A3.**

We agree with the reviewer that Bexarotene, in addition to antagonizing HBV/HDV entry by suppressing surface NTCP expression levels, is also expected to antagonize conjugated bile salts uptake into hepatocytes. To clarify this point in our manuscript, we analyzed the effect of Bexarotene on bile salts uptake function of NTCP. To use the most physiologically relevant in vitro model, we utilized the primary human hepatocytes (PXB). We added Panel G to figure 7 showing the suppression of sodium dependent bile salts uptake into primary hepatocytes pretreated with Bexarotene. We have no information on how surface NTCP transport is regulated in mouse because all our experiments in the manuscript were performed using human cells. Hence, the only way to respond to the reviewer comment on analyzing the effect of Bexarotene on bile salts uptake in vivo was to use human hepatocytes chimeric mouse. Unfortunately, we were unable to comply with this request due to the extremely high cost required for human hepatocytes chimeric mouse experiments which is far beyond our granting ability. However, we used the in vitro human primary hepatocyte as mentioned above, which is the closest in vitro option to the physiological condition.

To answer the reviewer comment

We added

**Results (lines 270 to 274)** we added the underlined text below:

**RXR agonists down-regulate KIF4 expression and block HBV entry by FOXM1 -mediated suppression**

Bexarotene, in addition to antagonizing HBV/HDV entry by suppressing surface NTCP expression levels, is also expected to antagonize NTCP-mediated conjugated bile salts uptake into hepatocytes. To clarify this point, we analyzed the effect of Bexarotene on sodium dependent bile salts uptake in primary human hepatocytes. As expected, Bexarotene significantly suppressed the bile salts uptake (p<0.05) (Fig. 7G).

**Figures**

We added Panel 7G

**Figure Legends**

We added **(lines 853 to 864)** we added the underlined text below:

**((G)** Primary human hepatocytes pretreated with DMSO or different concentrations of Bexarotene (1 μM, 10 μM, and 50 μM) for 72 h were incubated with [^3^H]-taurocholic acid at 37°C for 15 min in a sodium-containing buffer. After washing, the cells were lysed and intracellular radioactivity was measured and plotted as fold changes, relative to control DMSO-pretreated cells (*black bars*). Sodium dependent [^3^H]-TCA uptake was assessed in DMSO-pretreated cells either in the absence (negative control) or presence (positive control) of 100 nM Myr-B. Sodium independent [^3^H]-TCA uptake was also evaluated in DMSO-pretreated cells by incubation with [^3^H]-TCA in a sodium-free buffer (*gray bar*). All assays were performed in triplicate and data from 3 independent experiments were included. For panel **(G)**, the assay was performed in quintuplicate (n=5). The data were pooled to assess the statistical significance. Data are presented as mean ± SD. *, *P* < 0.05; **, *P* < 0.01; ***, *P* < 0.001.)

**Material and Methods**

**Lines 524 to 530** we added the underlined text below:

(NTCP transporter assay

NTCP bile transporter activity has been evaluated in primary human hepatocytes as previously described (45). Briefly, the cells pretreated with DMSO or Bexarotene for 72 h were incubated with [^3^H]-taurocholic acid in either sodium-containing or sodium-free buffer at 37°C for 15 min to allow [^3^H]-TCA uptake into the cells. After washing to remove free [^3^H]-TCA, the cells were lysed and intracellular radioactivity was measured using an LSC-6100 liquid scintillation counter.)

**Discussion, lines 377 to 379** the highlighted text below was added:

(Bexarotene, in addition to the suppression of HBV/HDV entry, also suppressed sodium dependent NTCP-mediated uptake of bile salts into primary hepatocytes (Fig. 7G).)

**Reviewer #2 Q4.**

Since the discovery of NTCP as the high affinity receptor for HBV/HDV, many studies have identified compounds that can inhibit viral entry, including the most well-known one, Myrcludex. From the data of Fig. 7, it’s apparent that the RXR agonist Bexarotene inhibits HBV/HDV infection to a much lesser extent than MyrB (Figs. 7 and 8) at a much higher dose (10 uM Bexarotene vs. 100 nM MyrB) in vitro. Furthermore, unlike MyrB that interacts directly with NTCP to block HBV/HDV interaction, RXR agonist works through downregulating the expression of FOXM1, a transcription activator of KIF4. Therefore, RXR agonist affects not only the expression of FOXM1 and KIF4 but also other downstream genes of RXR, which may result in more side effects irrelevant to HBV/HDV entry inhibition than MyrB. From the clinical point of view, RXR agonists seem not to be good drug candidates for inhibiting HBV/HDV entry. The authors should discuss these concerns and compare its clinical utility with other HBV/HDV entry inhibitors, in treating HBV infection.

**Reviewer #2 A4.**

To comply with reviewer’s comment, we changed **discussion** as following

**Lines 386 to 417** we added the underlined text below:

(Myrcludex-B is a lipopeptide and is therefore difficult to administer orally, the availability of other orally administrated drugs may increase drug feasibility and ease of use. Several compounds were reported so far to suppress NTCP-mediated HBV/HDV entry: Vanitaracin A, irbesartan, ezetimibe, and ritonavir, which directly block NTCP transporter activity, reduce LHBs-dependent viral infection (14-16) (17, 18); while Epigallocatechin-3-gallate (EGCG) and Ro41-5253 decrease cell surface expression of NTCP and reduce NTCP-dependent HBV infection (19, 20). Bexarotene belongs to this group of compounds reported to suppress NTCP surface expression, and showed the highest suppressive effect on HBV infection compared to EGCG and Ro41-5253 (Table 1). Although Bexarotene suppressed the transcription factor FOXM1, and KIF4 which may result in more side effects irrelevant to HBV/HDV entry inhibition, we did not find any cytotoxic effect of Bexarotene on hepatocytes using concentrations over 50 µM (CC_50_ > 50 µM) in our in vitro experimental system. Bexarotene is also approved by FDA for the treatment of cutaneous lymphoma. Furthermore, the IC_50_ of Bexarotene for suppressing HBV infection (IC_50_ 1.89 ± 0.98 μM) is much lower than that reported to induce suppression of cancer cells (IC_50_ 50-152 μM) (21, 22). Because HBV entry inhibition reduces the intrahepatic cccDNA pool (23), entry inhibitors are expected to be useful in preventing de novo infection in clinical settings such as vertical transmission and HBV recurrence post-LT. While further in vivo data are still required to assess efficiency and safety of NTCP targeting drugs including Bexarotene, the available data suggest their probable future use as prophylactic treatment for HBV infection. HBIG is used to inhibit HBV vertical transmission (5). Furthermore, extended therapy with HBIG in conjunction with a nucleos(t)ide analog is necessary following liver transplantation (LT) to reduce the HBV recurrence rate to less than 10% in 1-2 years post-transplantation (24). HBV-DNA integration into the host genome is known to be a risk factor for cancer development (25) and is reported to occur in the early weeks after infection (26, 27). An efficient prophylactic approach using combination of drugs which inhibits HBV cell entry (NTCP surface expression inhibitors, and NTCP-preS1 interaction direct blockers), might be beneficial as an alternative to HBIG to efficiently suppress NTCP-mediated HBV infection and prevent HBV-related complications like HBV-DNA integration and HBV-induced liver cancer. These data strongly suggest that Bexarotene and its functional analogs would be studied further for the development of a new class of anti-HBV agents.)

**Reviewer #2 Q5.**

In the discussion section, the contents of the paragraphs 2, 3 and 4 are largely overlap those in the results section. These contents should be re-organized to a more concise form. Moreover, the proposed role of KIF4 in the current HBV/HDV entry model should be included in the discussion and better be depicted in a diagram.

**Reviewer #2 A5.**

We followed the reviewer comment, and adjusted the discussion section as follows

**Lines 310 to 324**

(KIF4 belongs to the kinesin superfamily (KIFs). KIFs are ATP-dependent microtubule-based motor proteins that are involved in the intracellular transport (28). The N-terminal motor domain of KIF4 is in charge of ATP hydrolysis and microtubule-binding, whereas the C-terminal domain attaches to cargo molecules such as proteins, lipids, and nucleic acids (29). KIF4 has previously been implicated in the anterograde transport of cellular proteins such as Integrin beta-1 (30), as well as viral proteins such as retroviral (human immunodeficiency virus (HIV-1), murine leukemia virus, Mason-Pfizer monkey virus, and simian immunodeficiency virus) Gag polyprotein (31) to the cell surface to allow for efficient retroviral particle formation. In line with KIF4’s previously described anterograde transport function, we discovered that inhibiting the ATPase motor activity of KIF4 substantially reduced surface and raised cytoplasmic NTCP levels, a phenomenon that was accompanied by a significant suppression of HBV infectivity (Fig. 5). These findings demonstrated that KIF4 controlled the anterograde transport of NTCP to the cell surface, influencing its availability as a receptor for HBV/HDV entry on the hepatocyte surface.)

**The proposed role of KIF4 in the current HBV/HDV entry model was summarized in a diagram** in figure 9.

**Figures**

We added Figure 9

**Figure Legends**

We added **(Lines 896 to 904)**

**Proposed model for KIF4 role in NTCP-mediated HBV/HDV entry and impact of KIF4 targeting on HBV/HDV infection.** *Left panel*, KIF4 expression is regulated by transcriptional activator Forkhead box M1 (FOXM1) where cytoplasmic KIF4 pool utilizes microtubules to transport NTCP to hepatocyte cell surface where it can act as entry receptor for HBV and HDV. *Right panel*, Pan-RXR agonist, Bexarotene, downregulates FOXM1 and its down-stream KIF4 expression leading to impaired NTCP surface transport and suppression of HBV/HDV entry into human hepatocytes.

**Discussion, lines 370-372** the highlighted text below was added

our data clearly showed that the main mechanism by which Bexarotene suppressed HBV infection is through the downregulation of surface NTCP levels prior to HBV infection (Fig. 9).

**Part III – Minor Issues: Editorial and Data Presentation Modifications**

**Reviewer #2: Minor comments:**

**Reviewer #2 Q6**

Page 4, line 84: Because nucleocapsid cannot infect hepatocytes, the sentence “When HBV nucleocapsid infects human hepatocytes, it is carried to the nucleus” should be better changed to “When HBV infects human hepatocytes, its nucleocapsid is carried to the nucleus”.

**Reviewer #2 A6**

This comment is shared by reviewer one, we apologize for this mistake.

**Lines 83,84,** We changed to (When HBV infects human hepatocytes, its nucleocapsid is carried to the nucleus)

**Reviewer #2 Q7**

Page 19, line 352: “pruritis” seems to be a typo for “pruritus”.

**Reviewer #2 A7**

Pruritis was changed to pruritus on **Line 382**

**Reviewer #2 Q8**

Fig. 2, only HBsAg and viral DNA data were shown to illustrate the effect of KIF4 knockdown on HBV infection. It’s better to include HBe data in this figure because HBeAg secretion but not HBsAg secretion has been shown to correlate with HBV infection efficacy in the in vitro infection system. (Dr. Urban’s study, Gastroenterology, 2014; 146: 1070-1083)

**Reviewer #2 A8**

This comment is also shared by Reviewer 1 and the Editor. Please review our answer to Reviewer 1, **(**lines 61 to 170 **in this letter)**. This answer includes all the changes performed in the manuscript (experiments to detect HBeAg and HBV cccDNA).

**Reviewer #2 Q9**

In Fig. 7, the authors applied immunofluorescence staining of alpha-tubulin and co-localization analysis to demonstrate the interaction of KIF4 and NTCP over microtubule filaments. To make the result more solid, detection of microtubule-associated proteins, such as alpha-tubulin, in the KIF4 and NTCP co-immunoprecipitated complex is suggested.

**Reviewer #2 A9**

We followed the reviewer’s important comment, and we successfully detected microtubule-associated protein (alpha-tubulin), in the KIF4 and NTCP co-immunoprecipitated complex.

To reflect this finding in the Manuscript

We added **in Results** lines 225 to 239

(The co-immunoprecipitation study confirmed the interaction between NTCP and microtubule associated KIF4. Myc-tagged KIF4 and HA-tagged NTCP expressing vectors were co-transfected into HEK293-FT cells. We only noticed NTCP and the microtubule protein,α-tubulin co-immunoprecipitation when we used Myc antibody to pull down Myc-tagged KIF4, but not when we used control-IgG (Fig. 6B). To further investigate the interaction between these two proteins, we prepared recombinant KIF4 and NTCP proteins, after co-incubation of these proteins together, we performed a co-immunoprecipitation experiment by pulling down the recombinant Myc-tagged KIF4. Despite the successful pulling down of KIF4, we could not detect the co-precipitation of the recombinant NTCP protein (Fig. 6C). Overall, our findings suggest that KIF4 possibly binds indirectly to NTCP through a complex formation across the microtubules in the cytoplasm utilizing its ATPase motor domain to transport NTCP to the cell surface. In fact, KIF4 was previously reported to regulate the surface expression of L1 adhesion molecule through the participation in the antiretrograde transport of vesicles containing L1 to the axon shaft of neuronal cells on the microtubules (13).)

**Figures**

We added the detection of α-tubulin to Fig. 6B.

**Figure Legends (Lines 795 to 804)** we added the underlined text below:

**Fig. 6**

**((B)** HEK 293-FT cells were co-transfected with HA-NTCP, and Myc-KIF4 plasmid vectors (at a ratio of 1:1). At 3 days post-transfection, the cells were lysed and an aliquot of the cell lysate (1/10 volume) was used for the detection of Myc-KIF4 (*Input; uppermost panel*), HA-NTCP (*Input; upper middle panel*), α-tubulin (*Input; lower middle panel*), and β-actin (loading control) (*Input;* *lowermost panel*) by immunoblotting. The remaining cell lysates (9/10 of the original volume) were subjected to co-IP using either isotype control antibody or anti-Myc IgG to pull down Myc-KIF4. Following IP, each sample was analyzed by immunoblotting for Myc-KIF4 (*IP; upper panel*) and both co-immunoprecipitated HA-NTCP (*IP; middle panel*) and α-tubulin (*IP; lower panel*). All assays were performed in triplicate and data from 3 independent experiments were included.)

**Material and Methods, we added**

**Co-Immunoprecipitation (Co-IP) assay (Lines 569 to 571)**

Following IP, the samples were subjected to immunoblotting to detect co-immunoprecipitated HA-NTCP and α-tubulin (microtubule marker) in the pull-down fraction.

**Immunoblot assay (Line 537)**

anti-α-tubulin (Sigma, T5168),

**Reviewer #2 Q10**.

EGFR has been also been shown to be a druggable host-entry cofactor (PNAS, 2019; 116: 8487-8492). However, the authors did not find EGFR in their functional screening. This reviewer suggests to include this issue in the Discussion section.

**Reviewer #2 A10**. We followed the reviewer’s comment, and added in discussion, **Lines 325 to 338** we added the underlined text below:

(One of the selection criteria in our screening for pro- or anti-HBV host genes is that a significant effect should be detected upon silencing the expression of this gene with a minimum of two independent siRNAs targeting different sequences of its mRNA. Although EGFR is previously reported as an important druggable host-entry cofactor triggering HBV internalization (32), in our screening, a significant effect was detected with only one EGFR-targeting siRNA sequence, hence it was excluded from our selection. Because si-RNAs targeting different genes achieve favorable knockdown at variable concentrations, and the difficulty in assessing the optimum concentration for each si-RNA utilized in our high throughput screening, we performed the initial screening using siRNA at 10 nM concentration as recommended by the manufacturer, in contrast to 30- 100 nM concentrations utilized by Iwamoto *et al.* to silence EGFR expression. Hence, we expect that the discrepancy in the results regarding the effect of EGFR silencing on HBV infection is most probably attributed to the variation in the silencing efficiency of anti-EGFR siRNA utilized at different concentrations during transfection.)

**Reviewer #2 Q11**.

Fig. 7B, the signal intensity of each band should be quantitated as in Fig. 4.

**Reviewer #2 A11**.

We followed the reviewer’s comment

The signal intensity of surface NTCP was shown in Supplementary figure 2B.

To reflect these changes to the manuscript.

We added **in results**, **Lines 260 to 261**

Signal intensity is shown in Fig. S2B.

**Supplementary Figures**

We added supplementary Figure 2B

**Figure Legends**

Supplementary Figure 2B was added **Lines 920 to 923**

**(B)** The intensities of surface NTCP bands (normalized to CDH-1) shown in Fig. 7B were quantified by ImageJ software and presented as fold changes relative to the control DMSO treated cells.

**Reviewer #3:**

**Reviewer #3: Part I - Summary**

Sameh A. Gad et al report in this manuscript the discovery and mechanistic study of kinesin KIF 4 in HBV/HDV infection of hepatocytes. Through a siRNA screen of host cellular genes essential for the early events of HBV infection (from receptor binding to pgRNA production), the authors found that pgRNA reporter was significantly reduced when knockdown of KIF4 expression. The critical role of KIF4 in HBV infection was confirmed in HepG2-NTCP cells and PHHs. Mechanistic studies showed that knockdown of KIF4 significantly (i) reduced the level of preS1 peptide binding, (ii) compromised HDV infection, (iii) reduced NTCP cell surface localization. The authors further showed that the reduced NTCP cell surface expression in KIF4 knockdown cells can be restored by expression of WT, but not ATPase-null KIF4. Finally, the authors showed that treatment of cells with RXR-specific agonist bexarotene inhibited HBV and HDV infection, which also reduced cell surface NTCP expression.

Overall, the data presented strongly support a role of KIF4 in NTCP cell surface trafficking, which is consistent with the well known function of KIF4 in the anterograde transport of cellular and viral proteins to cell surface. Experimentally, several key issues need to be addressed.

**Reviewer #3:  Part II – Major Issues: Key Experiments Required for Acceptance**

**Reviewer #3 Q1**.

It is important to perform a complementation assay in KIF4 knockdown cell to show that expression of WT, but not the ATPase-dead KIF4, can restore HBV and HDV infection efficiency.

**Reviewer #3 A1**

This is a very important comment that was **shared by reviewers 1 and 3**.

Please review our answer, experiments and text modifications described above in this letter in our response to the second comment of Reviewer 1, **lines 177 to 275 in this letter**.

**Reviewer #3 Q2**.

It is curious that 10 μM Bexarotene treatment of HepG2-NTCP cells for 72 h does not alter the levels of total cellular KIF4 in the experiment presented in Fig. 7B, but reduced the level of total cellular KIF4 in the experiment shown in Fig. 7C. Explain?

**Reviewer #3 A2**.

We apologize because our data was unclear. **We did not show any expression of KIF4 in figure 7B,** we showed that Bexarotene did not affect the levels of total cellular NTCP (Input; upper panel) but suppressed surface NTCP level (Surface; upper panel).

**Reviewer #3 Q3**.

Song et al. showed that bexarotene inhibits HBV infection in HepG2-NTCP cells, HepaRG cells, and primary Tupaia hepatocytes, mainly through modulation of cellular lipid metabolism. In this report, the authors hypothesize that bexarotene reduces the level of transcription factor FOXM1, which is essential for KIF4 transcription. Although the data presented in Fig 7C showed that bexarotene treatment did reduce FOXM1, but the role of FOXM1 in KIF4 expression and NTCP cell surface trafficking remain to be experimentally determined in hepatocytes.

**Reviewer #3 A3**.

This is a very important comment that was **shared by reviewers 2 and 3**.

Please review our answer, experiments and text modifications described above in this letter in our response to the first comment of reviewer 2, **lines 376 to 431 in this letter**.

**Reviewer #3:  Part III – Minor Issues: Editorial and Data Presentation Modifications**

**Reviewer #3 Q4.**

**Reference 16 and 36 are duplicates**

**Reviewer #3 A4,**

We apologize for the mistake. References were corrected.

**NB**

**The References here are numbered based on their appearance in this letter. They carry different numbering in the manuscript.**

1.	Ibrahim MK, Abdelhafez TH, Takeuchi JS, Wakae K, Sugiyama M, Tsuge M, et al. MafF is an antiviral host factor that suppresses transcription from Hepatitis B Virus core promoter. J Virol. 2021.

2.	Watashi K, Liang G, Iwamoto M, Marusawa H, Uchida N, Daito T, et al. Interleukin-1 and tumor necrosis factor-alpha trigger restriction of hepatitis B virus infection via a cytidine deaminase activation-induced cytidine deaminase (AID). J Biol Chem. 2013;288(44):31715-27.

3.	Gudima S, Meier A, Dunbrack R, Taylor J, Bruss V. Two potentially important elements of the hepatitis B virus large envelope protein are dispensable for the infectivity of hepatitis delta virus. J Virol. 2007;81(8):4343-7.

4.	Fukano K, Tsukuda S, Oshima M, Suzuki R, Aizaki H, Ohki M, et al. Troglitazone Impedes the Oligomerization of Sodium Taurocholate Cotransporting Polypeptide and Entry of Hepatitis B Virus Into Hepatocytes. Front Microbiol. 2018;9:3257.

5.	Watashi K, Sluder A, Daito T, Matsunaga S, Ryo A, Nagamori S, et al. Cyclosporin A and its analogs inhibit hepatitis B virus entry into cultured hepatocytes through targeting a membrane transporter, sodium taurocholate cotransporting polypeptide (NTCP). Hepatology. 2014;59(5):1726-37.

6.	Endow SA, Kull FJ, Liu H. Kinesins at a glance. J Cell Sci. 2010;123(Pt 20):3420-4.

7.	Wu G, Chen PL. Structural requirements of chromokinesin Kif4A for its proper function in mitosis. Biochem Biophys Res Commun. 2008;372(3):454-8.

8.	Allweiss L, Volz T, Giersch K, Kah J, Raffa G, Petersen J, et al. Proliferation of primary human hepatocytes and prevention of hepatitis B virus reinfection efficiently deplete nuclear cccDNA in vivo. Gut. 2018;67(3):542-52.

9.	Gaunt CM, Rainbow DB, Mackenzie RJ, Jarvis LB, Mousa HS, Cunniffe N, et al. The MS Remyelinating Drug Bexarotene (an RXR Agonist) Promotes Induction of Human Tregs and Suppresses Th17 Differentiation In Vitro. Front Immunol. 2021;12:712241.

10.	Song M, Sun Y, Tian J, He W, Xu G, Jing Z, et al. Silencing Retinoid X Receptor Alpha Expression Enhances Early-Stage Hepatitis B Virus Infection In Cell Cultures. J Virol. 2018;92(8).

11.	Hu G, Yan Z, Zhang C, Cheng M, Yan Y, Wang Y, et al. FOXM1 promotes hepatocellular carcinoma progression by regulating KIF4A expression. J Exp Clin Cancer Res. 2019;38(1):188.

12.	Laoukili J, Kooistra MR, Bras A, Kauw J, Kerkhoven RM, Morrison A, et al. FoxM1 is required for execution of the mitotic programme and chromosome stability. Nat Cell Biol. 2005;7(2):126-36.

13.	Peretti D, Peris L, Rosso S, Quiroga S, Caceres A. Evidence for the involvement of KIF4 in the anterograde transport of L1-containing vesicles. J Cell Biol. 2000;149(1):141-52.

14.	Kaneko M, Watashi K, Kamisuki S, Matsunaga H, Iwamoto M, Kawai F, et al. A Novel Tricyclic Polyketide, Vanitaracin A, Specifically Inhibits the Entry of Hepatitis B and D Viruses by Targeting Sodium Taurocholate Cotransporting Polypeptide. J Virol. 2015;89(23):11945-53.

15.	Blanchet M, Sureau C, Labonte P. Use of FDA approved therapeutics with hNTCP metabolic inhibitory properties to impair the HDV lifecycle. Antiviral Res. 2014;106:111-5.

16.	Ko C, Park WJ, Park S, Kim S, Windisch MP, Ryu WS. The FDA-approved drug irbesartan inhibits HBV-infection in HepG2 cells stably expressing sodium taurocholate co-transporting polypeptide. Antivir Ther. 2015;20(8):835-42.

17.	Lucifora J, Esser K, Protzer U. Ezetimibe blocks hepatitis B virus infection after virus uptake into hepatocytes. Antiviral Res. 2013;97(2):195-7.

18.	Wang XJ, Hu W, Zhang TY, Mao YY, Liu NN, Wang SQ. Irbesartan, an FDA approved drug for hypertension and diabetic nephropathy, is a potent inhibitor for hepatitis B virus entry by disturbing Na(+)-dependent taurocholate cotransporting polypeptide activity. Antiviral Res. 2015;120:140-6.

19.	Tsukuda S, Watashi K, Iwamoto M, Suzuki R, Aizaki H, Okada M, et al. Dysregulation of retinoic acid receptor diminishes hepatocyte permissiveness to hepatitis B virus infection through modulation of sodium taurocholate cotransporting polypeptide (NTCP) expression. J Biol Chem. 2015;290(9):5673-84.

20.	Huang HC, Tao MH, Hung TM, Chen JC, Lin ZJ, Huang C. (-)-Epigallocatechin-3-gallate inhibits entry of hepatitis B virus into hepatocytes. Antiviral Res. 2014;111:100-11.

21.	Gretskaya NM, Gamisonia AM, Dudina PV, Zakharov SS, Sherstyanykh G, Akasov R, et al. Novel bexarotene derivatives: Synthesis and cytotoxicity evaluation for glioma cells in 2D and 3D in vitro models. Eur J Pharmacol. 2020;883:173346.

22.	Janakiram N, Steele V, Rao C. Combined effects of raloxifene (SERM) and bexarotene (Rexinoid) on growth suppression of colon cancer cells. Cancer Research. 2007;67(9 Supplement):1657-.

23.	Tu T, Urban S. Virus entry and its inhibition to prevent and treat hepatitis B and hepatitis D virus infections. Curr Opin Virol. 2018;30:68-79.

24.	Samuel D, Muller R, Alexander G, Fassati L, Ducot B, Benhamou JP, et al. Liver transplantation in European patients with the hepatitis B surface antigen. N Engl J Med. 1993;329(25):1842-7.

25.	Yang L, Ye S, Zhao X, Ji L, Zhang Y, Zhou P, et al. Molecular Characterization of HBV DNA Integration in Patients with Hepatitis and Hepatocellular Carcinoma. J Cancer. 2018;9(18):3225-35.

26.	Brechot C, Hadchouel M, Scotto J, Fonck M, Potet F, Vyas GN, et al. State of hepatitis B virus DNA in hepatocytes of patients with hepatitis B surface antigen-positive and -negative liver diseases. Proc Natl Acad Sci U S A. 1981;78(6):3906-10.

27.	Scotto J, Hadchouel M, Hery C, Alvarez F, Yvart J, Tiollais P, et al. Hepatitis B virus DNA in children's liver diseases: detection by blot hybridisation in liver and serum. Gut. 1983;24(7):618-24.

28.	Sheng L, Hao SL, Yang WX, Sun Y. The multiple functions of kinesin-4 family motor protein KIF4 and its clinical potential. Gene. 2018;678:90-9.

29.	Hirokawa N, Noda Y, Tanaka Y, Niwa S. Kinesin superfamily motor proteins and intracellular transport. Nature Reviews Molecular Cell Biology. 2009;10(10):682-96.

30.	Heintz TG, Heller JP, Zhao R, Caceres A, Eva R, Fawcett JW. Kinesin KIF4A transports integrin beta1 in developing axons of cortical neurons. Mol Cell Neurosci. 2014;63:60-71.

31.	Tang Y, Winkler U, Freed EO, Torrey TA, Kim W, Li H, et al. Cellular motor protein KIF-4 associates with retroviral Gag. J Virol. 1999;73(12):10508-13.

32.	Iwamoto M, Saso W, Sugiyama R, Ishii K, Ohki M, Nagamori S, et al. Epidermal growth factor receptor is a host-entry cofactor triggering hepatitis B virus internalization. Proc Natl Acad Sci U S A. 2019;116(17):8487-92.
