# Supplementary material for: The kinesin KIF4 mediates HBV/HDV entry through the regulation of surface NTCP localization and can be targeted by RXR agonists in vitro
Source: PLoS Pathog. 2022 Mar 21;18(3):e1009983. doi: 10.1371/journal.ppat.1009983 (PMC8970526; doi:10.1371/journal.ppat.1009983)
Supplement: Attachment — Submitted filename: Response to reviewers R1 Final.docx [file ppat.1009983.s004.docx]

**Re: PPATHOGENS-D-21-01953R1**

**Title: “The kinesin KIF4 mediates HBV/HDV entry through regulation of surface NTCP localization and can be targeted by RXR agonists in vitro.”**

Dear Dr. Jianming Hu:

Associate Editor (Plos Pathogens)

We wish to thank you, and our three reviewers very much for your time and effort you spent to improve this manuscript, and for your kind decision of minor revision. Enclosed we are sending our revised manuscript. In accordance with the reviewers’ suggestions, we have modified our paper and replied to the reviewers’ comments as shown below.

We believe that we have sufficiently revised our paper in accordance with the suggestions of the reviewers and that our revised manuscript is now acceptable for publication.

With our best regards,

Hussein H. Aly, and Takaji Wakita

National Institute of infectious diseases

1-23-1 Toyama, Shinjuku, Tokyo 162-8640, Japan.

Phone +81-3-5285-1111
FAX +81-3-5285-1150

e-mails: [ahussein@niid.go.jp](mailto:ahussein@niid.go.jp), and [wakita@niid.go.jp](mailto:wakita@niid.go.jp)

**Minor Issues: Editorial and Data Presentation Modifications**

**Reviewer 1**
**Comment 1**

I accidentally found a paper on the same topic by Hu et al entitled “E-cadherin plays a role in hepatitis B virus entry through affecting glycosylated sodium-taurocholate cotransporting polypeptide distribution” published in Frontiers in Cellular and Infection Microbiology in 2020 (10: 74). In that paper the authors claimed that E-cadherin promotes the cell surface localization of glycosylated NTCP to facilitate HBV infection of HepG2/NTCP cells, HepaRG cells, and PHHs. They found physical interaction between E-cadherin and glycosylated NTCP. Such findings are very similar to the relationship between KIF4 and NTCP. It remains to be seen whether both E-cadherin and KIF4 could modulate HBV infectivity through NTCP localization, or the report on E-cadherin is unreliable. For fairness of literature review this paper on the same topic should be cited and probably discussed briefly in the Discussion section.

**Answer 1**

First, we wish to thank you very much for your time, and suggestions to improve our manuscript. We apologize for missing the discussion about the role of E-cadherin in the localization of glycosylated surface NTCP. Based on the reviewer’ s comment, we added the following paragraph to the discussion section, and new references.

(Page 20; lines 365 to 376)

**Discussion**

E-cadherin was previously reported to facilitate HBV infection and the localization of glycosylated NTCP to the cell surface (1). In that report, a direct interaction between E-cadherin and glycosylated NTCP was found. However, the substantial role of this interaction and the mechanism of E-cadherin regulation of surface distribution of glycosylated NTCP was not investigated (1). E-cadherin is an essential component of adherens junctions and is required to establish cellular polarity (2). HBV infects human hepatocytes at the basolateral membrane (3), where NTCP is located exclusively (4). Hence, E-cadherin may affect HBV entry either through the regulation of hepatocyte polarization and NTCP surface distribution on the basolateral membrane, interacting with cell surface NTCP-HBV complex triggering HBV internalization into the cell, or through participation in NTCP surface transport. Further analysis is required to explore the function of E-cadherin in HBV entry.

**References**

**NB**

**The References here are numbered based on their appearance in this letter. They carry different numbering in the manuscript.**

1.	Hu Q, Zhang F, Duan L, Wang B, Ye Y, Li P, et al. E-cadherin Plays a Role in Hepatitis B Virus Entry Through Affecting Glycosylated Sodium-Taurocholate Cotransporting Polypeptide Distribution. Front Cell Infect Microbiol. 2020;10:74.

2.	Shneider BL, Fox VL, Schwarz KB, Watson CL, Ananthanarayanan M, Thevananther S, et al. Hepatic basolateral sodium-dependent-bile acid transporter expression in two unusual cases of hypercholanemia and in extrahepatic biliary atresia. Hepatology. 1997;25(5):1176-83.

3.	Schulze A, Mills K, Weiss TS, Urban S. Hepatocyte polarization is essential for the productive entry of the hepatitis B virus. Hepatology. 2012;55(2):373-83.

4.	Sun AQ, Swaby I, Xu S, Suchy FJ. Cell-specific basolateral membrane sorting of the human liver Na(+)-dependent bile acid cotransporter. Am J Physiol Gastrointest Liver Physiol. 2001;280(6):G1305-13.

**Reviewer #2:**

**Comment 1**

**I would suggest editing some parts of the “Discussion section” to achieve better logical flow and conciseness. For example, paragraph 3 (lines 340-346) seems to be overlapped a lot with the results and does not add too much insight for the discussion.**

**Answer 1**

We completely agree with the reviewer’s comment. To prevent redundancy, we removed the paragraph in question from the discussion section.

**Comment 2**

**There are English writing issues in the newly added texts. English editing is suggested.**

**Answer 2**

English Editing was performed. Edited sentences were underlined in the new manuscript.

**Comment 3**

**P19, lines 363-364, the author stated that “Bexarotene suppresses NTCP expression ---”. However, this manuscript and DR. Li’s work all showed that Bexarotene does not affect the expression of NTCP at total protein and RNA levels, respectively. Therefore, this sentence should be changed to “Bexarotene suppresses surface NTCP expression (or surface localization) ---”.**

**Answer 3**

We sincerely apologize for this mistake. (**Bexarotene suppresses NTCP expression) was modified to (Bexarotene suppresses NTCP surface localization) (Page 19 lines 354-355)**

**Comment 4**

**p31 line 573: 4C  4oC.**

**Answer 4**

We changed 4C to 4ºC. (Page 31, line 576)

Finally, we wish to thank the **Editor**, and **our three reviewers** for the good experience they gave us while processing our manuscript; for all the constructive comments that really improved the quality and scientific aspect of the work, and for their timely response.

Sincerely

Hussein H. Aly
